# 3D printed opto-microfluidic autonomous analyzer for photometric applications

**DOI:** 10.1016/j.ohx.2023.e00406

**Published:** 2023-02-28

**Authors:** Camarillo-Escobedo Rosa, Flores-Nuñez Jorge, García-Muñoz Luis, Camarillo-Escobedo Juana, Peña-Dominguez Edgar

**Affiliations:** a National Technological Institute of Mexico – La Laguna, Mechanic and Mechatronics Department, Blvd. Revolución & Calz. Cuauhtemoc S/N, Torreon, Coah., Mexico; b Universidad de Guadalajara-CUCEI, Translational Biomedical Engineering Department, Av. Revolución #1500, Guadalajara, Jal, Mexico; c National Technological Institute of Mexico – La Laguna, Computer System Department, Revolución & Calz. Cuauhtemoc S/N Torreon, Coah., Mexico; d National Technological Institute of Mexico – La Laguna, Electric and Electronic Department, Blvd. Revolución & Calz. Cuauhtemoc S/N, Torreon, Coah., Mexico

**Keywords:** Opto-microfluidic, Microanalyzer, Automation microprocesses, Auto-calibration, Optical detection, Hydrodynamic system

## Abstract

3D printed opto-microfluidic autonomous analyzer for photometric applications performs the automation of analytical micro-processes. The proposed device was designed under restrictions of small size and low energy consumption, which allow its portability for in-situ, on line and real time analysis. The autonomous process and auto-calibration consists of four functions: control and data acquisition; hydrodynamic: fluid pumping and flow injection; optical detection and wireless communication. All electronics systems where controlled with a virtual instrument interface. In the experiments carried out to measure fluorides, the results obtained were very close to those obtained with laboratory equipment. The consumption of reagents was 50% less and waste was reduced by 80%. The cost of the portable and autonomous microanalyzer is 75% less than large and robust laboratory equipment.

## Graphical abstract

**Figure:**
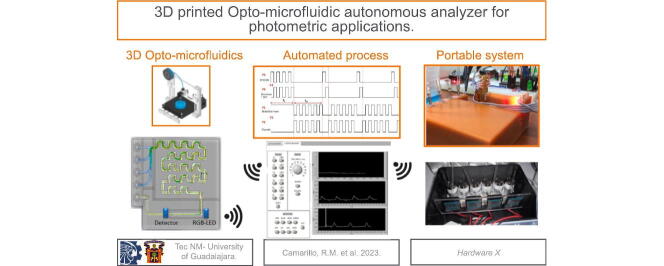

**Specifications table:** 

Hardware name	3D printed opto-microfluidic autonomous analyzer for photometric applications
Subject area	• Chemistry and biochemistry
Hardware type	• Field measurements and sensors
Closest commercial analog	*“No commercial analog is available.”*
Open source license	**OSHWA** (Open Source HardWare Association) CC BY 4.0
Cost of hardware	*$2,050.40 USD*
Source file repository	https://doi.org/10.17632/64ykjhczr6.3

## Hardware in context

Automation and miniaturization are trends for the development of analytical systems. At present, the design of these systems is based on constraints: a high performance of *in-situ* measurements, high selectivity, multi-sample analysis and devices highly adaptable to different analytical methods. With the miniaturization, one can achieve a significant reduction in the volume of reagents used and waste production during the procedure, and also an increment in the number of analysis samples per day by reducing the analysis time and cost [1], [2]. Terms Lab-on-a-chip and µTAS (micro total analysis) [3], [4], are considered synonyms for devices that work with fluids and integrate a number of different functionalities on the small scale [5], [6], designed for the implementation of analytical process: including sample preparation, transportation, mixing, separation, reaction, detection, signal acquisition and processing. These analytical systems are generally related to the sample volume of the process, not to the structure’s size [7]. The method of flow injection analysis (FIA), is one of the most used automated analysis method, due to its simplicity, versatility, low cost and its fast response time, so it can be used in monitoring systems [8], [9]. Microfluidics allows the possible to integrate multiple fluid tasks into a chip. The detection cell consists of the optical elements like lenses and waveguides and optoelectronic devices such as light sources, sensors, some ones could be kept off the chip. According to these characteristics, the opto-microfluidic systems provide significant advantages: high portability, efficiency, sensitivity, versatility, and low cost [10].

In biochemical and chemical analysis, usually, the samples are analyzed with two or more solutions or reagents. These solutions must be completely mixed for the chemical reaction to take place. In the opto-microfluidics structure, the mixture is developed into microchannels, reactors, heaters and sensing cells with very small Reynolds numbers. The mixed solutions are carried out mainly by diffusion assuming a laminar behavior. The small distances within the microfluidic channels enable complete mixing in a short time period, in the order of seconds. While in macroscale the mixture is achieved by turbulence.

Colorimetric and UV–VIS spectrophotometry techniques have been used in many analytical methods. They can be used for stand-alone or combined with separation/flow-through systems such as HPLC, FIA, electrophoresis or even portable point-of-care devices [11]. The development of portable and miniaturized devices based on colored reactions is an area still in development due to new materials, manufacturing techniques and an improvement in optoelectronic and electromechanical devices [12], not forgetting the novel computer design tools. Generally, microfluidic systems are manufactured using silicon [13], glass [14], [15], polymers [16], or LTCC (low temperature co-fired ceramics) [17] technologies, which require a sophisticated and very expensive infrastructure. For that technologies, the most of the studied cross-sections channels are limited to only rectangular and more recently trapezoidal shapes. Additive manufacturing technology is used as an alternative to those manufacture procedures [18], where one of the most common methods is the Fusion Deposition Modeling (FDM) [19], [20], also called “3D printing”.

## Hardware description

### Hardware overview

Instrumental methods for flow analysis have been evolving in order to carry out continuous control of a greater number of samples. The flow injection technique is generally the most used automated method. To carry out this instrumental method, large actuators are integrated, such as a peristaltic pump with an approximate cost of $1,580 and modular valve positioners with an approximate cost of $1,500, and to perform spectrophotometric analysis a Vis spectrophotometer with a cost of $4,437.44 and/or USB4000 spectrophotometer, cost $618. An analytics software application is typically priced at $226. The management of fluids using large volumes of reagents and the waste generated also generate an impact in the cost. Likewise, for determinations of low concentrations of metal ions, colorimetric methods, atomic absorption spectrophotometer ($10,000–$25,000), atomic emission spectrometer ($25,000–$33,000), etc. are integrated into the analytical process. Incorporating this type of equipment in instrumental methods of analysis has the following disadvantages. 1. The analytical process is required to be developed in central laboratories, making it impossible to implement it *in-situ* or on-line in the measurements. 2. The consumption of reagents as well as the production of waste is high, which is why it is expensive. 3. When performing off-line analysis, the information obtained is not in real time while performing the analysis. 4. They require trained human resources for the operation of this equipment in the analysis.

**Fig. 1:**
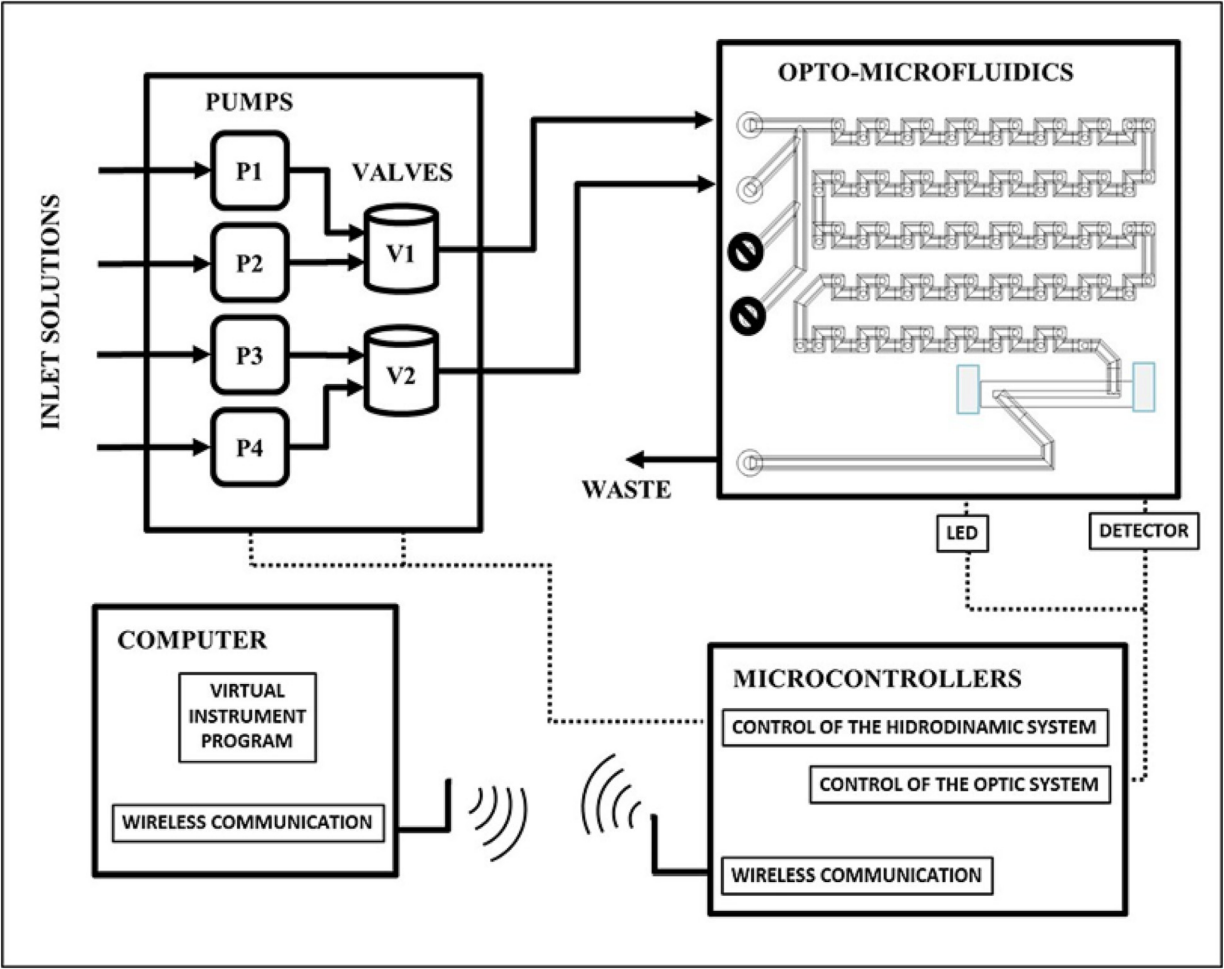
A block diagram of the proposed setup.

The proposed hardware of the 3D printed opto-microfluidic autonomous analyzer for photometric applications can be conceptualized in the following four functional modules: 1 Control and data acquisition, 2 Hydrodynamic system (fluid pumping and flow injection), 3 Optical detection system and 4 Wireless communication system. These modules were integrated to an opto-microfluidic structure were the analytic processes take place. Fig. 1 shows the block diagram of the basic elements of the design. The system was designed in modules looking its versatility and all operations should be developed by the control software (virtual interface). This modular design will allow the extension or modification of device without the need for major changes. The opto-microfluidic autonomous analyzer consists of four solution inlets and, one waste outlet and a fluid circuit in an area of 80 mm × 67 mm with a width of 1.5 mm. While, the detection section consists of a light source, a sample cell with an optical path of 30 mm and a photo-detector array. The handling of fluids in the fluidic circuit and the optical detection system are controlled by a microcontroller with a virtual interface.

The miniaturization offered by this opto-microfluidic analyzer has the following characteristics:

• Fast response.
• Low reagent and power consumption for its operation.
• Autonomous, portable and easy-to-operate by Wireless communication.
• Micro-Analyzes *in -situ*, on-line and in real time.
• Low cost $2,050.40 USD

The integration of micro-devices and opto-microfluidic structures to perform the mixing between solutions and optical detection produces a robust, flexible, portable and low cost analytical microsystem.

The proposed system that is described in this work presents a modular integration and automation of the analytical process not reported in other 3D printed devices developed for similar applications. The main characteristics of operation and design of the 3D printed devices developed were compared with the performance of the 3D Opto-microfluidics autonomous analyzer. This is shown in Table 1.

**Table 1:** Analytical features and main characteristic of operation of 3D printing devices [18].

**Description**	**Material**	**Detection system**	**Flow system**	**Analyte**	**Sample**	**Automated meso TAS**	**Ref**
3D photometric detector flow cell	PLA	Photodiode, multicolour LED (RGB)	–	Orange G.	–	–	[21]
3D LOV	Acrylic transparent resin	GF-AAS (Lab.Eq.)	MSFIA	–	Cd, Pb water	–	[22]
3D smartphone- spectrophotometer	PLA (only for holders)	White LED, collimator, cuvette	–	Cristal violet	–	–	[23]
3D flow cell for spectrophotometry	PLA	Photoresistor, Green LED	–	Nitrite	–	–	[24]
3D Printed Opto-microfluidic *	PLA, PP	64 Photodiodes matrix, RGB LED	Multi-commutation of microactuators	Methylene blue, Methyl red	Fluoride water	Auto-calibration, automated analytical method	[18]

* Proposed system in this work.

## Design files summary

**Table:** 

**Design file name**	**File type**	**Open source license**	**Location of the file**
1-Control	Design.PDSPRJ	CC BY 4.0	“Available with the article”.
2-Drivers micropumps	Design.PDSPRJ		“Available with the article”.
3-Power microvalves	Design.PDSPRJ		“Available with the article”.
4-Microfluidic structure	Design.STL		“Available with the article”.
5-Regulator bank	Design.PDSPRJ		“Available with the article”.
6-Article	The source of this dataset		https://www.sciencedirect.com/science/article/abs/pii/S0924424722000632

## Bill of materials summary

**Table:** 

**Designator**	**Component**	**Number**	**Cost per unit -currency**	**Total cost – currency**	**Source of materials**	**Material type**
3- Power microvalves: This are the micro-actuators to volume control	Solenoid isolation valves, 3 way NC, 161T030	6	66.81 USD	400.86 USD	https://www.nresearch.com/	
3- Power microvalves: these are the hydraulic connections	Barb fittings, port 10–32, barb 1/8, FITM082, polypropylene	12	0.50 USD	6.00 USD	https://www.nresearch.com/	Polymer
3- Power microvalves: these are the hydraulic connections	Silicone tubing, ID 1/8, OD 1/4, TBGM103	2	3.10 USD	6.20 USD	https://www.nresearch.com/	Polymer
2-Drivers micro-pumps	Micropumps, SDMP-306	6	136.68 USD	820.08 USD	https://www.takasago-fluidics.com/	
2-Drivers micro-pumps	Controller board MPD-200A	3	136.47 USD	409.41 USD	https://www.takasago-fluidics.com/	
4-Microfluidic structure	3D Microfluidics structure	1	1000 MXP	1000 MXP	tecsol3D.com	Polymer
4- Microfluidic structure: these are the hydraulic connections	Fitting of titanium ID = 1.5 mm, OD = 2 mm	5	100 MXP	500 MXP	W/R	Metal
4- Microfluidic structure: these are the optical cell window	*Borosilicate glass microscope slides	1/100	9.78 MXP	9.78 MXP	https://www.sigmaaldrich.com/	Ceramic Composite
1-Control	Kingbright SMD/SMT LED Emitters	10	0.54 USD	5.4 USD	https://www.mouser.com/	Semiconductor
1-Control	TAOS TCS3200 Sensor	1	4.47 USD	4.47 USD	https://www.electronicoscaldas.com/	Semiconductor
1-Control	Microcontroller, 18F4550	1	7.85 USD	7.85 USD	https://www.mouser.com/	Semiconductor
1-Control	Oscillator circuit 4 MHz	1	0.37 USD	0.37 USD	https://www.digikey.com.mx/	
3-Power micro-valves	Bipolar Transistors – BJT SMD	5	0.5 USD	2.50 USD	https://www.mouser.com/	Semiconductor
1-Control	Capacitors SMD 1 µf 22 pf 10 pf Electrolytic 1 µf	9 2 5 1	0.55 USD 0.41 USD	9.35 USD 0.41 USD	https://www.mouser.com/	Ceramic Aluminum
1-Control	Standard LEDs - SMD Water Clear Green 525 nm Blue 450 nm Yellow 570 nm Red 720 nm	2 6 1 1	0.68 USD	8.84 USD	https://www.mouser.com/	Semiconductor
1-Control	Trimmer -Resistors SMD 5kΩ	2	1.83 USD	3.66 USD	https://www.mouser.com/	Ceramic
1-Control	MAX232ACWE	1	7.30 USD	7.30 USD	https://www.mouser.com/	Semiconductor
1-Control	Resistors SMD 12.1 kΩ 10 kΩ 1 kΩ	5 3 6	0.35 USD	7.0 USD	https://www.mouser.com/	Ceramic
5-Regulator bank	Solid-state voltage regulators SMD, 3.3Vcd	1	0.59 USD	0.59 USD	https://www.mouser.com/	Semiconductor
5-Regulator bank	Solid-state voltage regulators SMD, 5Vcd	4	0.89 USD	3.56 USD	https://www.mouser.com/	Semiconductor
1-Control	Modules Bluetooth LM-058	2x adpt	198.15 USD	198.15 USD	https://mexico.newark.com/	
1-Control	Battery ion-Li, VSO-F550H	1	15.99 USD	15.99 USD	https://www.battdepot.com/	
1-Control	Regulated eliminator ELI-2500	1	439 MXP	439 MXP	https://www.steren.com.mx/	
full system storage cabinet	Plastic cabinet 15 × 10 × 5.5 cm	2	349 MXP	698 MXP	https://www.steren.com.mx/	Plastic

*Only one slide of the borosilicate glass microscope pack was used (1/100).

## Build instructions

The developed hardware has four functional modules whose details are described below.

### Control and data acquisition

**Fig. 2:**
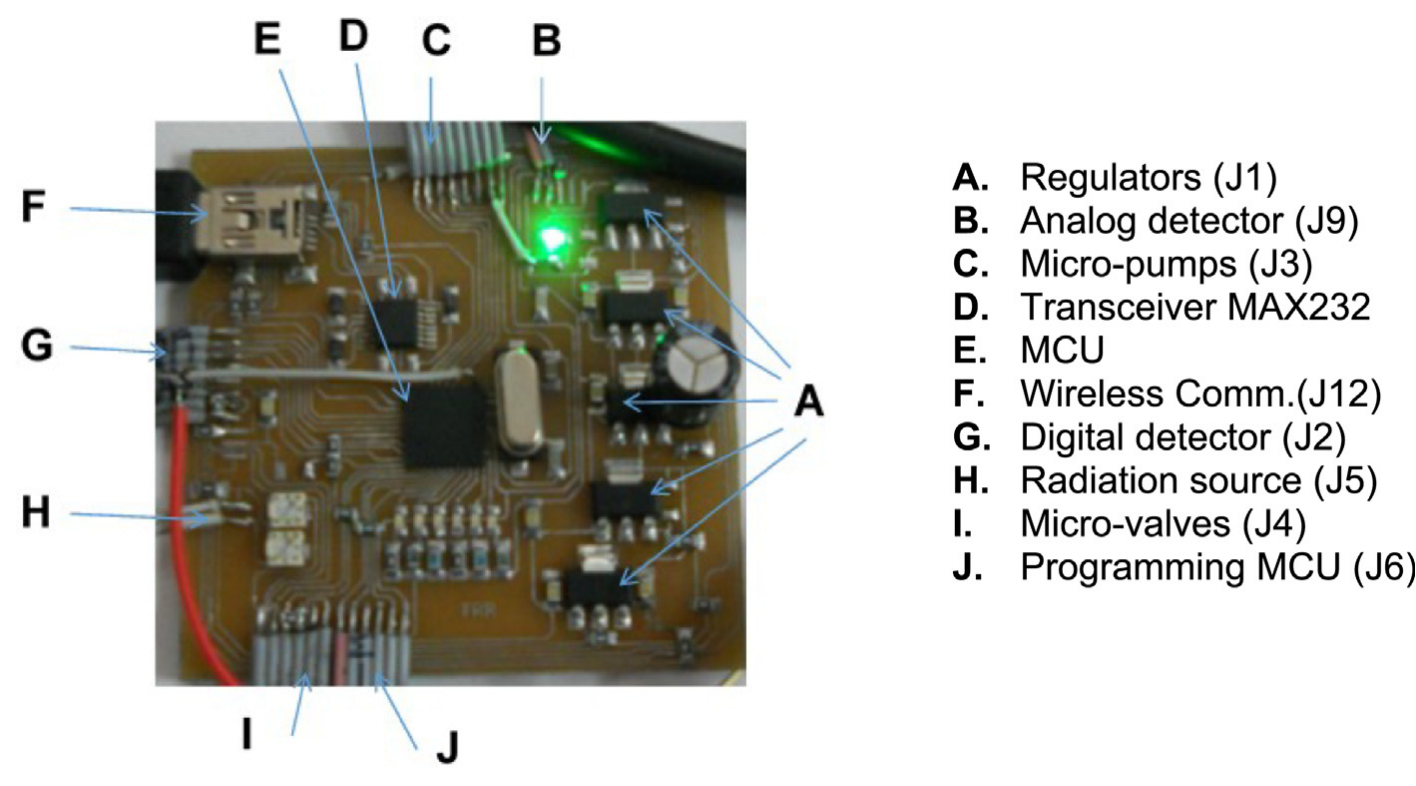
Electronic control module.

The developed electronic control and data acquisition was implemented in a compact PCB module 50 × 50 × 10 mm low cost manufactured by JLCPCB (jlcpcb.com, China). The electronic design consist of a 10-bit ADC with 1 analogic input, and 19 digital outputs, see Fig. 2. The system was designed for control and data acquisition of an automated analytical process. The module was designed with the versatility of performing analytical processes for other photometric measures just by re-programming the control module without the need for major hardware changes. The analytic process was configured and operated by the virtual program. The module includes the electronic control for data acquisition, flow sequence, and a wireless communication system. The control was implemented with a PIC18F4550/QFN44 microcontroller unit MCU (Microchip Technology Inc., Chandler, USA). To enable the portability of the system, it was fed with a lithium-ion rechargeable battery VSO-F550H (Amstron PS, Valencia, USA) of 7.2 V–2.2 A/h and a regulated eliminator ELI-2500 from 3 to 12 Vdc, 2.5 A (Steren, USA) as second source. Wireless communication between the device and control electronics consisted in Bluetooth modules LM-058 (LM Technologies, London, UK). A regulated bank was integrated into the control electronics to maintain the independent supply to different devices avoiding fluctuations to obtain a correct measurement.

#### Power supply

**Fig. 3:**
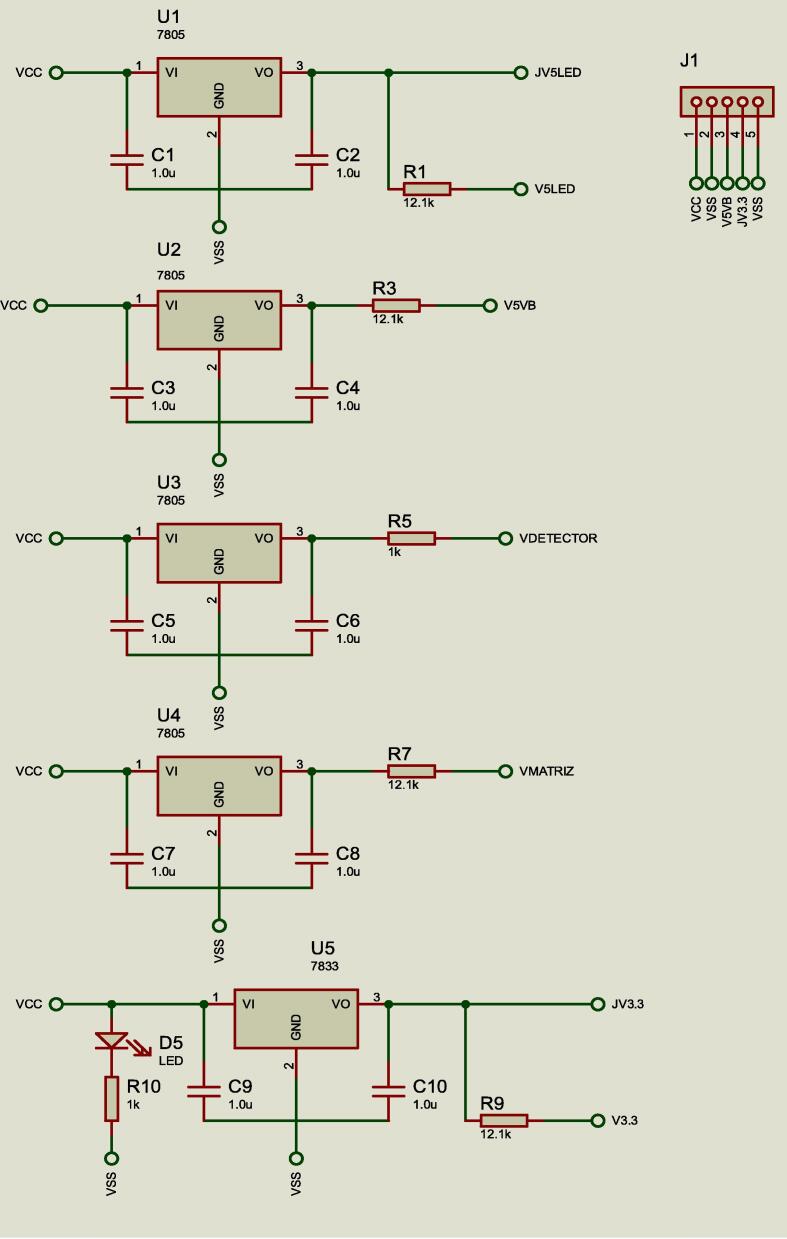
Regulation bank circuit.

The power supply consists of two power sources: an ELI-2500 regulator and a VSO-F550H battery with a flyback type topology. The voltages delivered by the source are 5 V and 3.3 V referred to a common ground (**VSS**). The power supply is regulated by 5 solid-state voltage regulators as shown in Fig. 3. **U5** regulates the power to the MCU at 3.3 V; **U1** feeds a TRIM adjusted (voltage divider) at 5 V as a radiation intensity control of LED to avoid over saturation of the photodetector; **U2** provides a regulated 5 V voltage to power the micro-pump drives, the wireless module and the saturation common of the micro-valve power stage transistors. **U3** supplies a regulated 5 V to the photo-detector and **U4** supplies 5 V to the multi-spectrum array.

In photometry, the light absorbed by a sample is a function concentration and optical path. Therefore, fluctuations in the light intensity of the beam can affect the measurements. For these reason, the light source should be stabilized in intensity emitting a continuous beam of light at specific wavelength. In general, the light intensity fluctuations can be due to variations in voltage or current, so it is powered independently and with a voltage regulation system. The independence of the regulators **U1**, **U3** y **U4** is important to ensure a stable voltage that will produce a stable light intensity.

#### Digital output

**Fig. 4:**
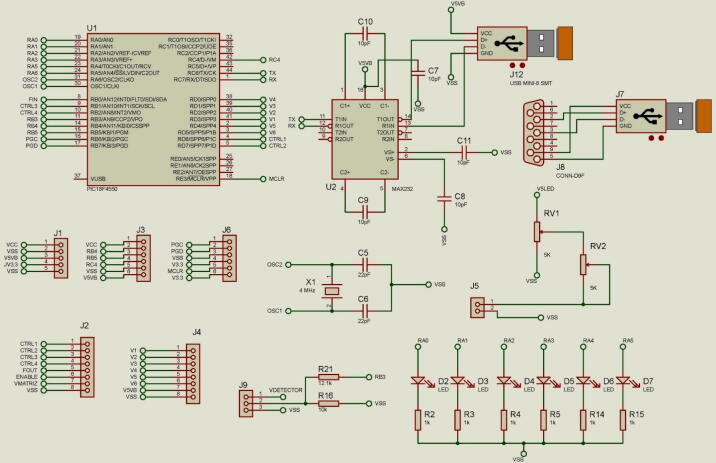
Control and acquisition electronic diagram.

The control provides 19 digital outputs as shown in Fig. 4. 12 outputs to control the micro-valves (**RA0**-**RA5** and **V1**-**V6**); 4 to control the multi-spectrum matrix (**CTRL1**-**CTRL4**) and 3 to control the micro-pumps (**RB4**, **RB5**, **RC4**). The digital outputs have a light indicator to show the status of the micro-valves.

#### Analog input

**Fig. 5:**
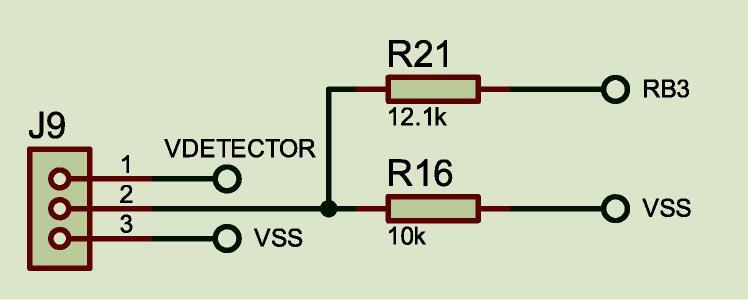
Photodetector coupling circuit.

The control provides 13 analog inputs. Fig. 5, depicts a connector block were we used only the **RB3** signal for the acquisition of the analytical signal photodetector through connector **J9**. **VDETECTOR** is voltage out coming from a voltage regulator 5 V which supplies the photodetector.

#### PWM signals

For the acquisition of the signal originating from the multi-spectrum sensor, the **FIN** input was used as shown in Fig. 4. This discrete signal has a frequency range from 200 Hz to 7.5 KHz, which works with a MCU timer to quantize the operating frequency.

The PWM signal are generated by the MCU timers for the control of the micro-pumps which have an operating frequency of 0 Hz to 60 Hz to generate flow rates up to 7.5 mL/min. Fig. 4 shows the PWM signal **RC4, RB4** and **RB5**, these modulated signals are applied to the MPD-200A micro-pump driver control for its operation.

#### Wireless communication

For communication a serial port was used. The transmission speed of this port is configurable into the range from 1.2 to 115,200 bauds. Through **J12** with a mini-usb interface port, the data are received and sent through the **TX** and **RX** control lines as see in Fig. 4. The communication port is based on the RS232 protocol with “Half Duplex” communication.

#### Maintenance and programming

The maintenance stage includes the necessary connection for program updates through a programmer Pickit^TM^ 3 (Microship Tech., USA). In the programming of the MCU, the PIC-C software was used. Fig. 4 depicts the signals **MCLŔ/VPP, VDD, VSS, PGD (ICSPDAT), PGC (ICSPCLK)** and **PGM (LVP)** for programming.

#### Connectors description

**J1** as shown in Fig. 4, connects the external power supply and the safety voltage output through **VCC** on pin 1 and **VSS** on pin 2, both voltajes are supplied by the ELI-2500 source. While Pin 3 with the nomenclature **V5VB** is the 5 V voltage output to the micro-pumps, in the same way pin 4 **JV3.3** is the 3.3 V voltage output that feeds the MCU and pin 5 **VSS** is the voltage reference or ground.

In Fig. 4 the connector **J2** was used to obtain signals from the TCS3200 detector. The **CTRL1** and **CTRL2** pins select the scaling of the sensor output signal; **CTRL3** and **CTRL4** select the group of photodiodes from the RGB array. **FOUT** is the signal processed by the MCU which is a frequency modulated signal with 50% duty cycles. **VMATRIX** is the 5 V supply voltage to the detector, which is an independent voltage to avoid electronic noise in the acquired analytical signal.

**J3** as shown in Fig. 4, connect the signals **RB4, RB5** and **RC4** from MCU that was used to the control drivers of the MPD-200A. The output voltaje in **V5VB** is used as power supply of 5 V for these drivers. Also through this connector, power is supplied to the entire system through **VCC** and **VSS**.

**J4** connector has the function of sending the control signals to the micro-valves through pins **V1, V2, V3, V4, V5** and **V6**. The 5 V power supply uses the **V5VB** and **VSS** for the power stage of the injection module.

**Fig. 6:**
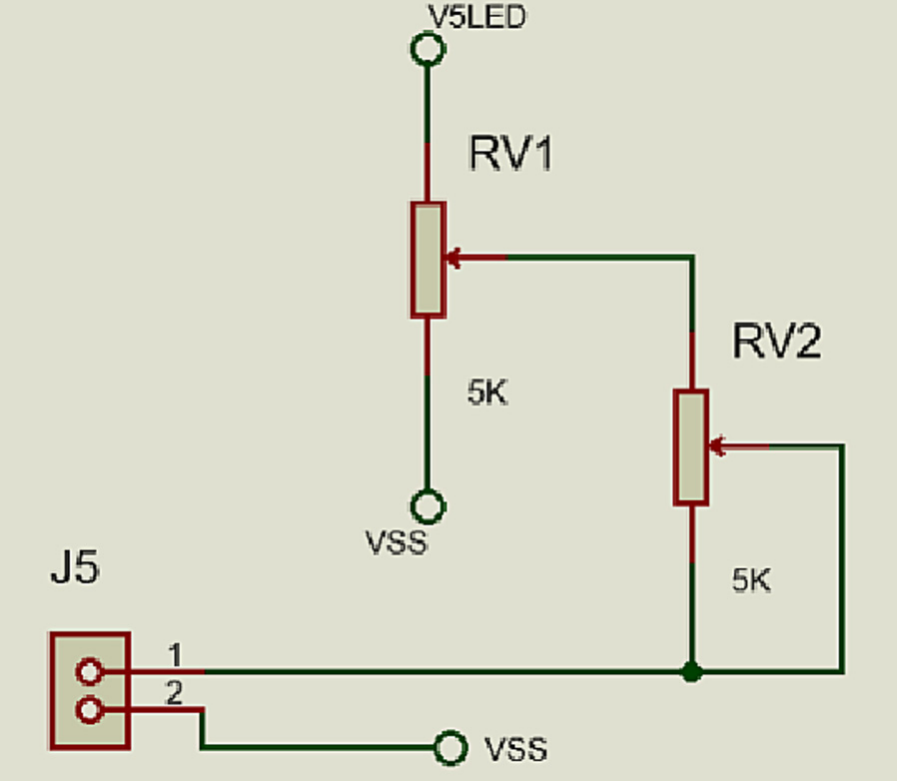
TRIM adjustment circuit.

**J5** connector controls the intensity of light source (micro-LED) through a TRIM adjustment composed of two potentiometers **RV1** and **RV2** allowing up to a maximum of 150 mA see in Fig. 6.

**Fig. 7:**
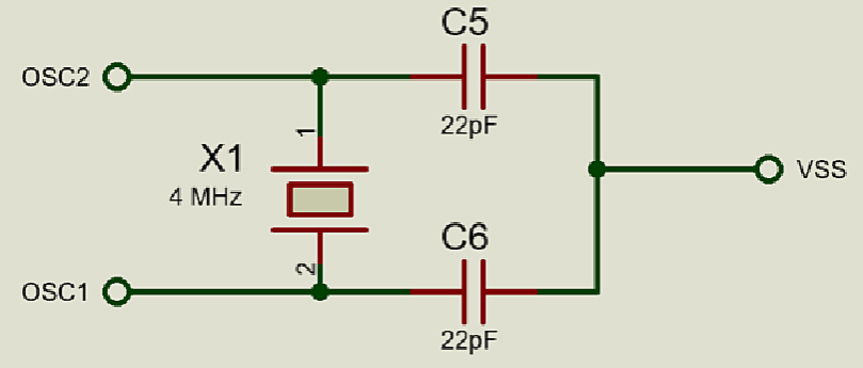
Oscillator circuit.

**J6** connector corresponds to the programming inputs of the MCU. It is a maintenance and firmware update port. The MCU uses a high-speed crystal at 4 MHz to obtain a very high precise and stable frequency of oscillators. An arrangement of 22pF capacitors was implemented to **VSS** for the management of the timers. The electronic diagram is shown in Fig. 7.

It is recommended to place a decoupling ceramic capacitor at the power supply inputs of the MCU between the 3.3 V and **VSS** lines.

**Fig. 8:**
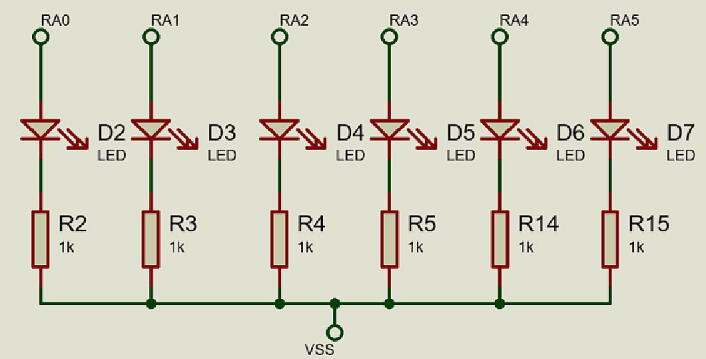
LED’s microvalves monitor.

In the control and data acquisition module, six micro-LEDs were integrated to monitor the states of the micro-valves, to show if the signal that activates the valves was established through wireless communication, which does not interfere with the LEDs, nor with the micro-valves. The micro-LEDs used have a dimension of 0.8 × 1.2 × 0.25 mm with a clear water lens, a operating current consumption of 20 mA and a emitting wavelength at 470 nm (Kingbright, USA). Its electronic diagram is shown in Fig. 8.

### Hydrodynamic module

#### Fluid pumping module

For the continuous flow system piezoelectric micro-pumps (Takasago Electric, Inc., Nagoya, Japan) were used, with dimensions 25 mm × 25 mm × 4.8 mm. All external connecting tubes were silicone tubing (NResearch, N.J., USA) with a 1.5 mm internal diameter. The micro-pumps were controlled by MPD-200A controller (Takasago Electric, Inc., Nagoya, Japan). Each controller can operate 2 micro-pumps.

Micro-pumps can be controlled by frequency or voltage. For this device, frequency control was selected. The MPD-200A controller can generate a range of frequencies from 1 Hz to 60 Hz with a maximum flow rate of 9 mL min^−1^ and minimum flow rate of 650 µL min^−1^. Three MPD-200A controllers with dimensions of 30 mm × 30 mm × 18 mm are connected as well as the VSO-F550H battery. Through **J3** connector there is communication interface with the control and acquisition module. The control is done through the **RC4, RB4** and **RB5** pins of the MCU which send frequency modulated signals to the MPD-200A.

**Fig. 9:**
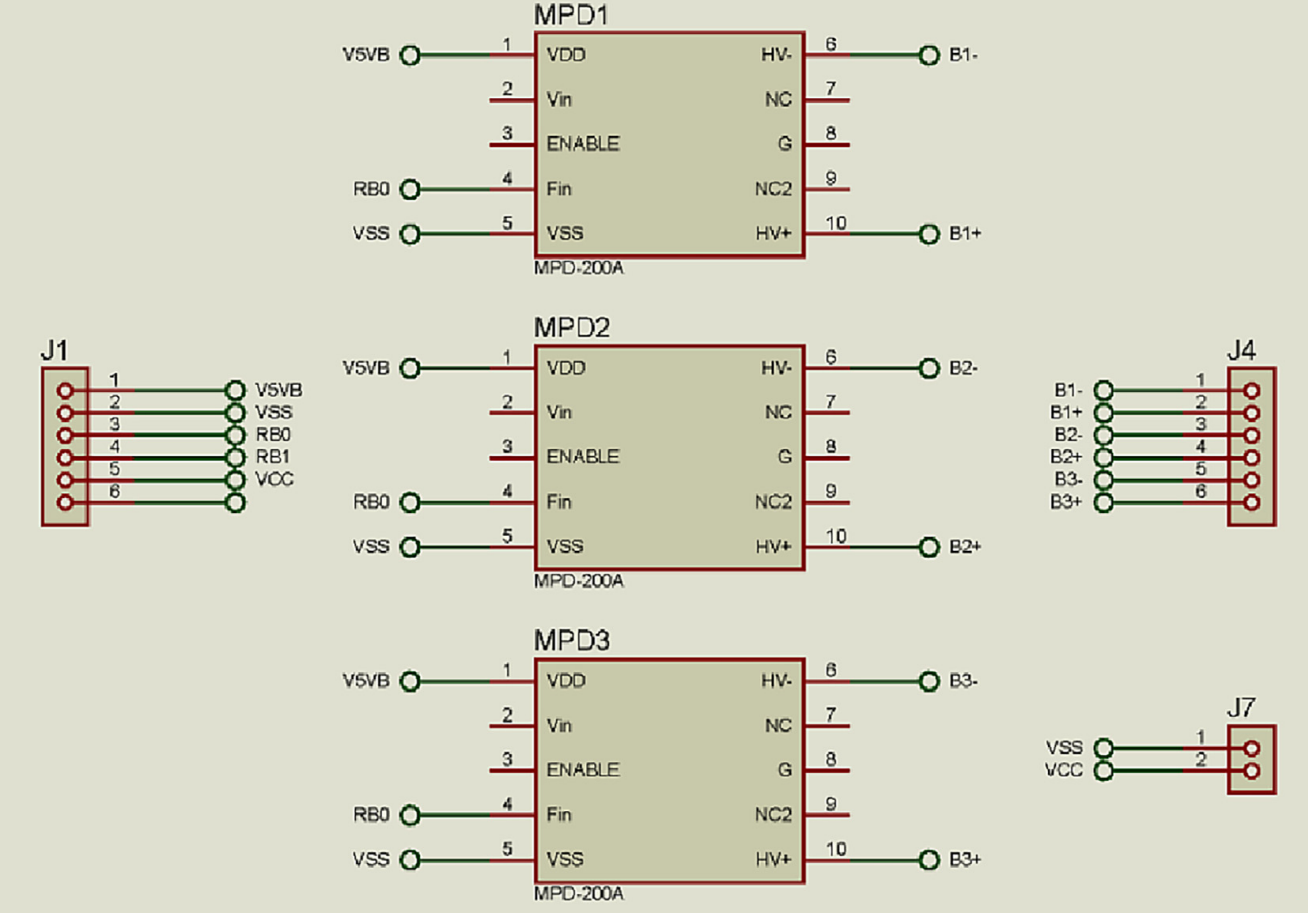
Driver pumping electronic diagram.

In Fig. 9, one seen that **U4, U5** and **U6** correspond to 3 MPD-200A controllers, while **J1** connector corresponds to the communication with the control and acquisition module through its connector **J3**. **J4** connector corresponds to the connection of the pumps to their respective controllers and **J7** connects the VSO-F550H battery.

**Fig. 10:**
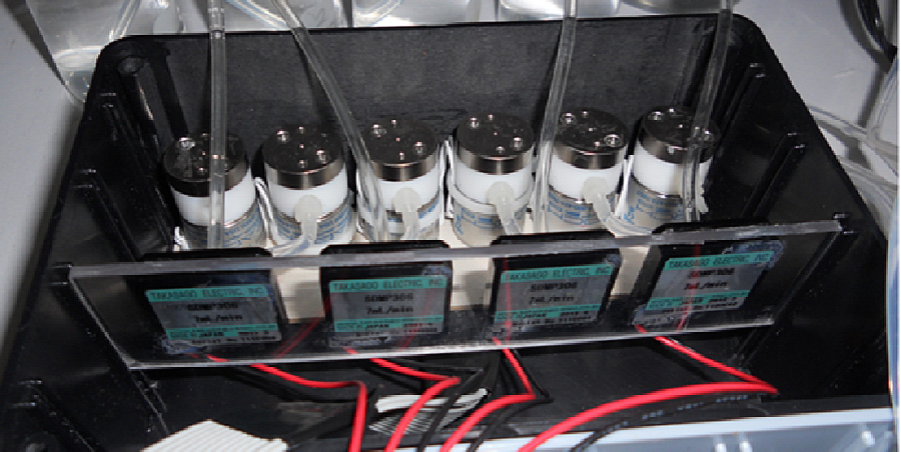
Flow injection module with micro-valves and micro-pumps assembly.

The pumping module is shown in Fig. 10. The micro pumps were fixed with epoxy glue on an acrylic sheet with a dimension of 14.5 × 3.5 cm.

#### Flow injection module

An injection system was designed by multi-commutation using three-way solenoid micro-valves (161T010, NResearch, NJ, USA). In order to develop a robust and flexible hydrodynamic system, a collector with 6 incorporated micro-valves was implemented for future analysis in case that a greater number of reagents will be involved. Each micro-valve can be interconnected with a micro-pump, so this hydrodynamic system has the capacity to manage 6 micro-valves for 12 solution outlet ports where each port can be configured to a different injection volume. Fig. 10 depicts the flow injected module.

**Fig. 11:**
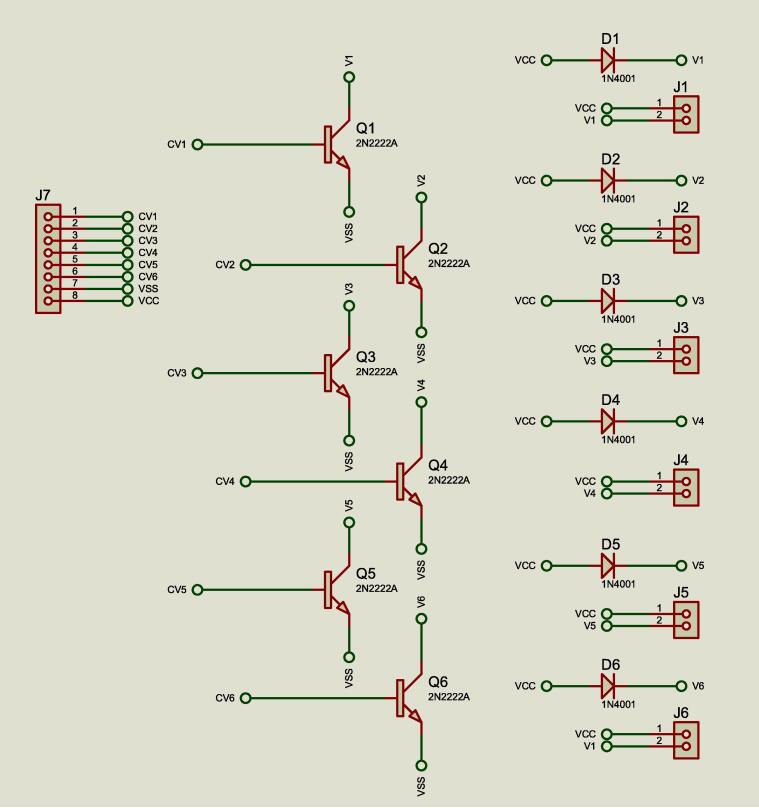
Power circuit for micro-valves.

The micro-valves are powered by 5 V, in multi-conmutation, considering an on time (open) of 20 ms and an off time (closed) of 30 ms. The power circuit was implemented with 6 NPN Bipolar Transistors – BCP56-16/SOT223/SC-73 (Nexperia, Guangdong) allowing a current collector-emitter, *Ice* = 500 mA. Each transistor energizes the micro-valves, it switched from **VCC** = 5 V and to **VSS** ground, voltage obtained from the control and acquisition module as shown in Fig. 11. **J7** connector receives the control signals from the control and acquisition module. From **J1** to **J6**, the power supply is connected to the micro-valves **V1, V2, V3, V4, V5** and **V6** and diodes **D1, D2, D3, D4, D5** and **D6**.

The function of the injection module is to control the flow (volume) of reagents and samples to the microanalyzer where the analytical process takes place.

### Optical detection module

The optical radiation source consists of an RGB LEDs module, size of 6 × 6 mm^2^ (Mod. AAA-505103, Kingbright Co., CA, USA). The packaged dimensions are 4 × 4 mm^2^ for lens and the 3 micro-LEDs distribution into de radiation area of the RGB LEDs is <2 mm^2^ with 120° of viewing angle [25]. The emission wavelengths are: for the blue LED λ = 450 nm with a spectral linewidth of 20 nm and optical power of 0.6 W; for the red LED λ = 624 nm, with a line width of 30 nm and an optical power of 0.336 W and for the green LED λ = 525 nm with a line width of 30 nm and an optical power of 0.6 W. In addition, a second LED module was integrated, with a size of 1.6 mm × 0.8 mm × 0.6 mm (KG DELLS1.22-JGKH-24, ams OSRAM, Regensburg, Germ.), whose emission wavelength is λ = 570 nm with a spectral bandwidth of 22 nm and optical power of 0.5 W. These modules can be replaced with different radiation sources according to the wavelength required for other colorimetric analysis, demonstrating its versatility. The light coupled into the sample cell is well homogenized and verified it with a spectrophotometer in the output window of the sample cell.

**Fig. 12:**
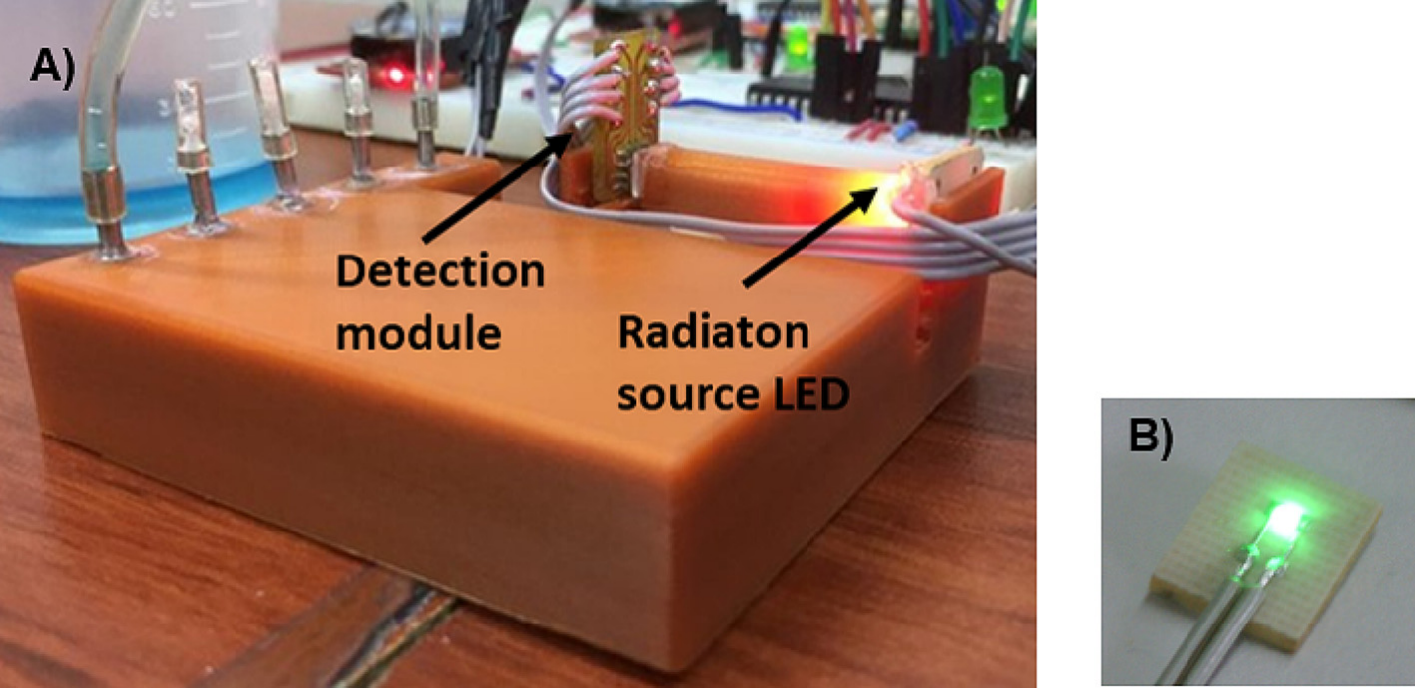
Optical detection module: A) Elements placed in microfluidic structure; B) Single LED module.

The detection module consists of a TAOS TCS3200 programmable light-to-frequency converter, (Texas Advanced Optoelectronic Solution TAOS, Texas, USA.) and dimension of 5 × 6.5 mm. This detector integrates an array of 64 photodiodes of which 16 photodiodes correspond to each color filter: red, green, blue (RGB) and another 16 photodetectors without filter (clear) for a greater detection spectral range. All four types of photodiodes are interdigitated to minimize non-uniformity of incident light radiation and are interspersed within the photoactive area. It is possible to select the degree of scaling of the obtained signal through an internal frequency divider that achieves frequency values ​​of 100%, 20%, 2% and off. The detector window has a diameter of 2.8 mm where the photodiode array with a photoactive area of ​​1 × 1 mm is located. The light source and detection module, was placed and integrated modularly on the microfluidic device as shown in Fig. 12.

**Fig. 13:**
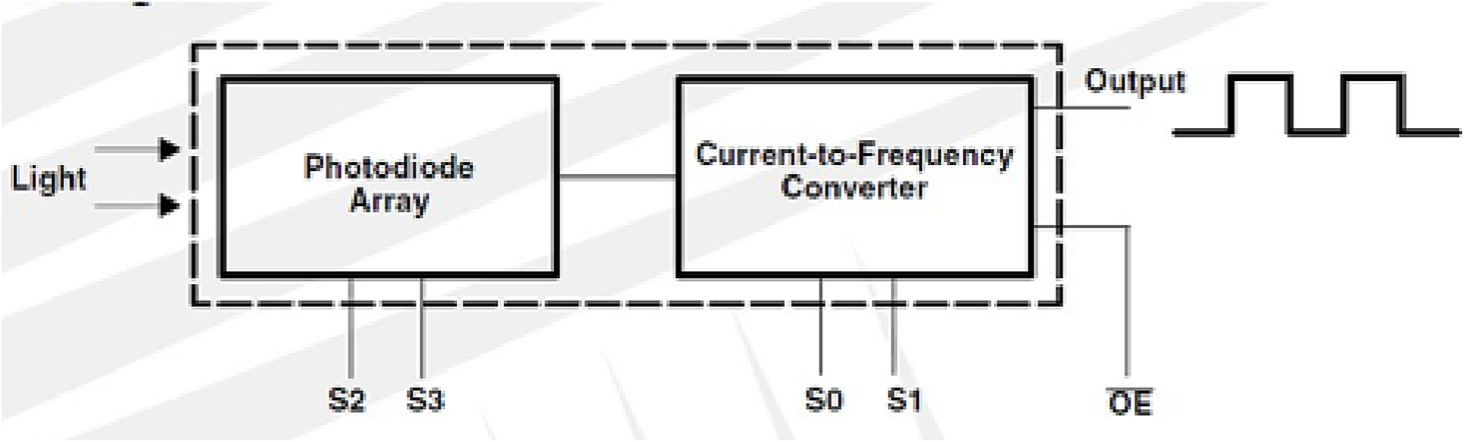
Functional block diagram of detector [26].

The output of the TAOS TCS3200 sensor is a 50% duty cycle square signal with a frequency proportional to light intensity. The full scale output frequency can be scaled by one of three present values through two control inputs. Digital inputs and digital outputs allow direct interface to the microcontroller or any other logic circuit. The output is put into a high impedance state to share this line to the microcontroller. The **S2** and **S3** inputs are used to select which group of photodiodes (blue, green, red, and white) will be actívated. While, **S0** and **S1** are used to select the scaling of the output signal, as well as to turn off the device. This type of logical operation makes it possible to implement it through control electronics. In Fig. 13 see a schematic diagram.

### Wireless communication

The transmission and reception of data between the hardware of the opto-microfluidic analytical system and the computer that processes the information was carried out by wireless communication using two LM-058 modules with Bluetooth protocol, with a communication range of up to 100 m, than that provided by wiring (maximum 12 m).

**Fig. 14:**
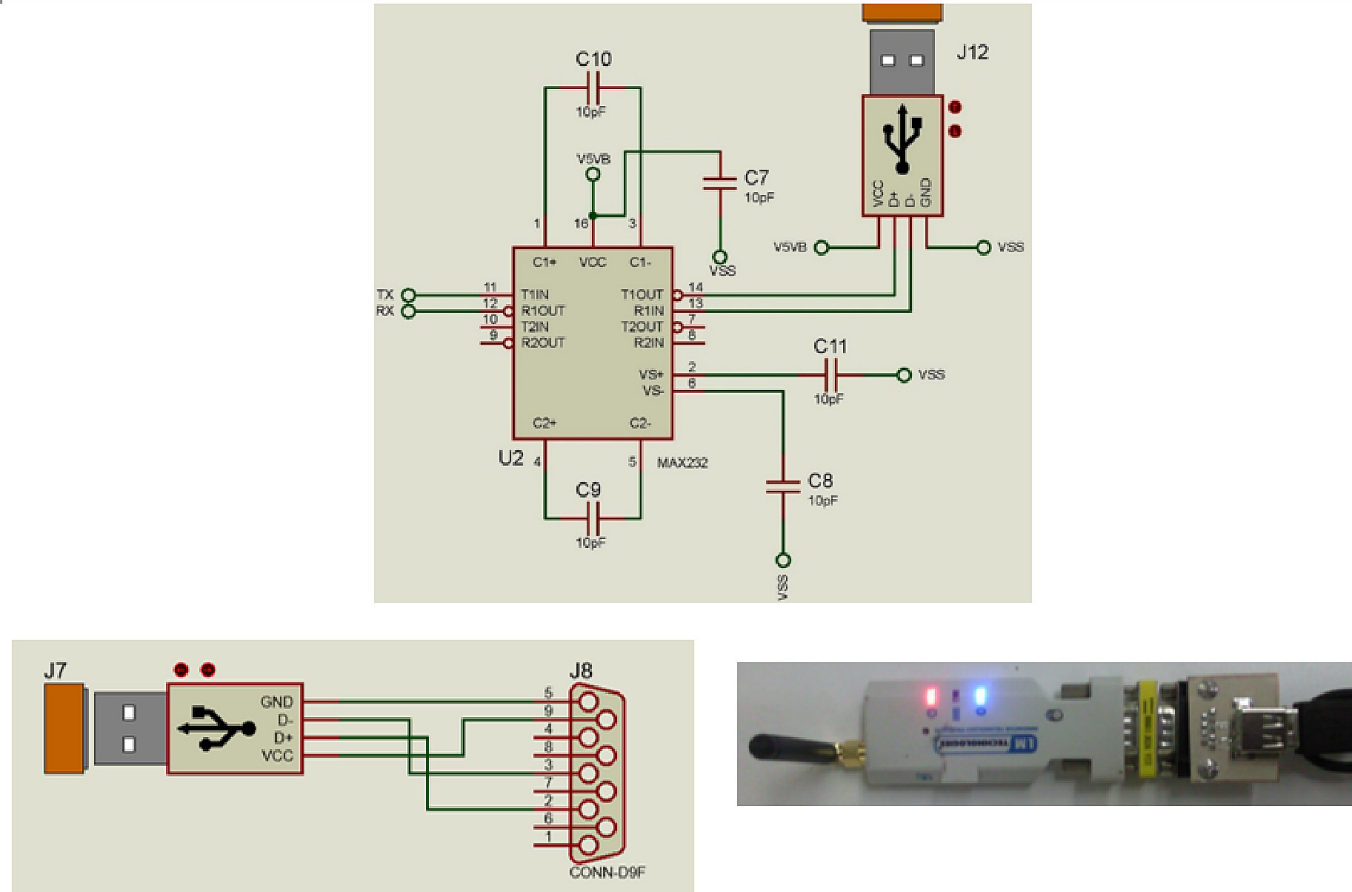
RS-232 Transceptor.

The wireless communication is focused on the automation of analysis through a graphical interface developed in a virtual instrument software. The main objective for the interface consists in the remote operation of the microanalyzer and the visualization of the analytical data. The link was point to point in an ad-oc network, where the PC is configured as master and the microanalyzer as slave. In Fig. 14, the electronic diagram shows the serial communications transceiver implemented in both modules: the control and acquisition, which establishes the communication between the devices, it converts the TTL voltages of the MCU to the RS232 protocol voltages. This transceiver is based on a MAX232ACWE + T (Maxim Integrate, Cal. USA). PIN 11 and 12 are the Tx and Rx signals of the MCU.

### Opto-microfluidic structure

**Fig. 15:**
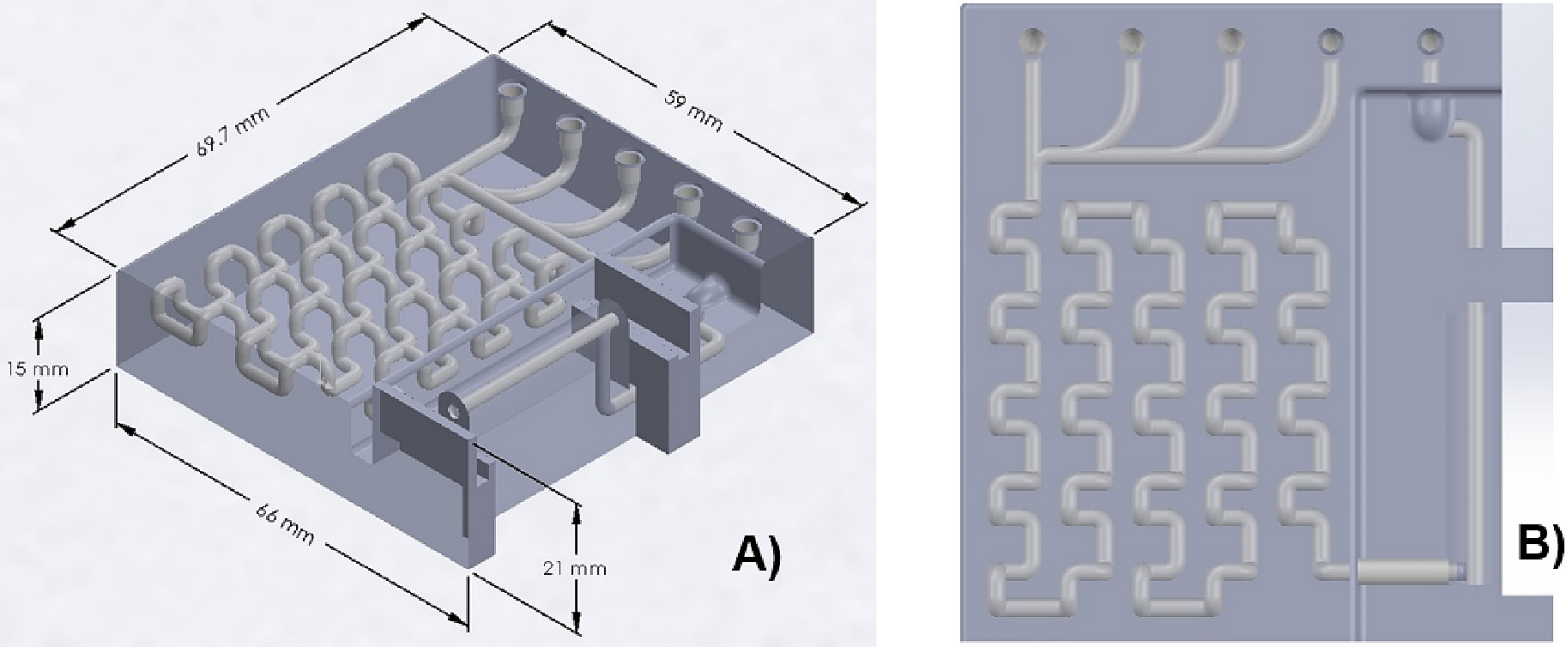
Design of microstructure: A) internal geometry, B) Cross-section channels.

The opto-microfluidic was manufactured used the fusion deposition modeling (FDM) technology also called “3D printing” and Polypropylene PP (TecSol3D, Monterrey, Mex.) was used as material. It is resistant to chemical solvents, bases and acids. The geometry of the opto-microfluidic device was designed in Solidworks®, which consisted of: a micromixer with circular 3D serpentine chaotic advection, a sample cell with a volume of 95 µL, four solution inlets and one waste outlet. The connectors for inlet and outlet fluids were made of titanium (MAFSA, Torreón, Mex.) with an internal diameter of 1 mm and an external diameter of 1.5 mm, them were fixed with epoxy glue applied on the surface of the top. The detection cell was integrated in the microfluidic device with an optical path length of 30 mm, and the windows were covered with a 0.12 mm thick borosilicate glass (Corning® cover glasses) (Sigma Aldrich, Naucalpan, Mex.) allowing light beam to pass through the cell. The borosilicate glass allows 90% transmittance whenever working at a wavelength between 0.4 and 2.0 μm, and it will not affect the absorbance measurements. The microfluidic device is shown in Fig. 15.

## Operation instructions

Once all its parts have been connected, according to Fig. 4, the following analytical considerations and set up operations are carried out.

• Select the colorimetric analytical method for the measurement of the analyte to be determined.
• Simplify and miniaturize the analytical method.
• Develop the timing diagram for injection sequence.
• Set point:
• Set the hydrodynamic values for the flow rate and injection volume according to requirements of the process.
• Set the working wavelength and calibrate the intensity of the radiation source by adjusting TRIM.
• Select the microvalves to operate according to the injection-timing diagram and feed the hydraulic ports with solutions and reagents.
• Run the virtual program for the automation process.
• Configuration.
• Automation.

For this application case, fluoride was determined.

### Analytical method

According to the Mexican standard NMX-AA-077-SCFI-2001 for the measurement of fluorides, the SPANDS method was used based on the reaction between fluoride ions and the colorful complex of Zirconyl-SPADNS. As the fluoride concentration increases, the intensity of the color decreases. Therefore, the absorbance is inversely proportional to the concentration of fluorides. The reaction was carried out in an acid medium. The stock solution was based on NaF 100 mg F^-^L^-1^, as reagent ZrCl_2_·8H_2_O (Zirconium chloride octahydrate) with concentrated HCl, and SPANDS solution. Fluoride standard solutions were prepared with concentrations of 0.5, 1.0, 1.5, 2.0 and 2.5 mg L^-1^. The colored solution from the reaction between fluoride ion and zirconyl acid-SPANDS reagent was measured at 570 nm. The single light module was selected because of the wavelength.

Analytical measurements were evaluated in terms of absorbance, which can be described as [27]

(1) $$A=\;-\;Log\;\left[\left(S_{a}-\;S_{d}\right)\;/\;\left(S_{r}-S_{d}\right)\right]$$

where *S_a_* is the analytical signal for the sample, *S_r_* is the reference signal (bi-distilled water) and *S_d_* is the noise or dark current signal.

### Set point

Before carrying out an analysis, it is necessary to perform a characterization of the modules that consists of setting operating parameters in order not to allow some variables to change that could alter the analytical measurement.

a) Optical detection module: For the detection system, the intensity of the light source (LED) was adjusted to 75 mW by a resistive adjustment TRIM system, obtaining an optimal value at 30 Ω and a maximum operating current of 50 mA.
b) Development of hydrodynamic variables: To simplify the operation, the micropumps are set at the same flow rate at *Q* = 3.9 mL min^−1^, (F = 13 Hz) in order to control the injection volume. If the flow rate is known, which represents volume per unit of time (speed) in the microchannel area, it is possible to define a desired injection volume. The injection operation of reagents and standard solutions was based the on–off time operation. Therefore, a time sequence for the valve operations was programed and then it was implemented in time function. The multi-commutation time for the injection was set at 100 ms.

### Auto-calibration processes

The auto-calibration process develops a series of operations to establish the relationship between the response of the microanalyzer and fluoride concentration (F), based on standard solutions, which leads us to define the operating range in which the microanalyzer will be able to perform measurements to determine the concentration of fluoride in a real sample.

The analysis involves the injection of two reagents, the standard solution and bidistilled water. According to the proposed method (miniaturized and simplified), the ratio of sample to reagents was 5:1.

The procedure for calibration consisted in the injection of a fluoride standard solution + bi-distilled water, selecting the microactuators P1, P2, V1. This process was executed by multi-commutation of V1, developed 0.5, 1.0, 1.5, 2.0 and 2.5 mg L^-1^ concentrations of standard solutions.

**Fig. 16:**
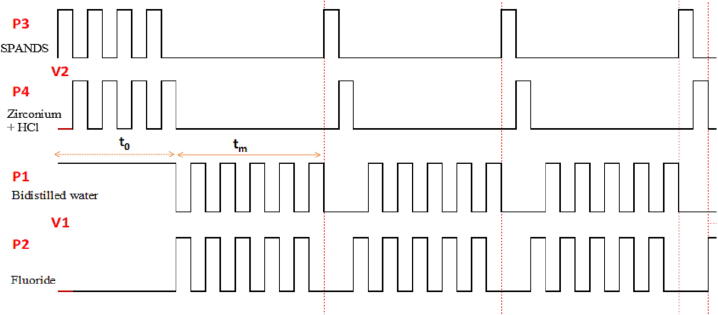
Timing diagram for injection sequence during the measuring of fluoride.

The SPANDS solution and the Zirconium + HCl reagent were injected selecting the microactuators P3, P4, V2. Then, the sample was injected at a 5:1 ratio between sample/SPAND + reactive in acid medium, generating volumes in the order of 500 µL/100 µL respectively. The timing sequence for the automated micro-analytical procedure is shown in Fig. 16 were t_0_ represents the time to generate the reference line or base line in V2 and t_m_ is the sampling time in V1.

**Fig. 17:**
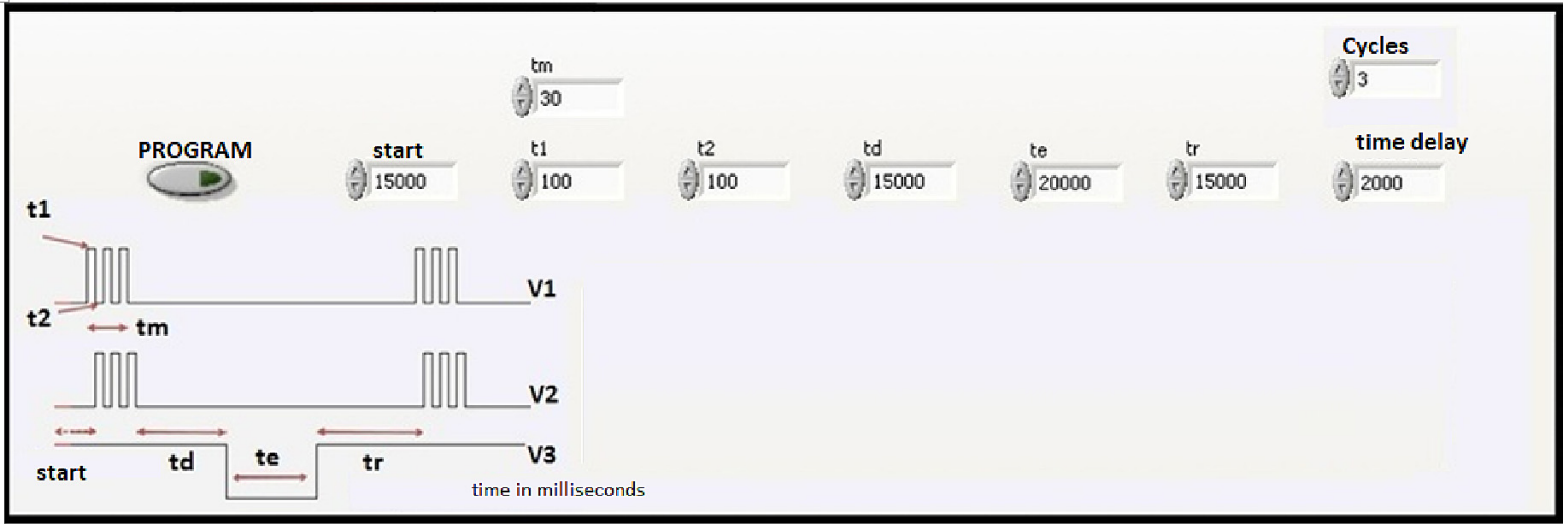
Frontal panel of virtual interface. Valves injection time program, where t_1_ is the turn-on time, t_2_ is the turn-off time, t_m_ is the sampling time, t_e_ is the residence time in the detector, t_d_ is the time it takes for the injected sample volume to reach the detection system, t_r_ is the time it takes to clean the lines and fill them with carrier solution or baseline. Cycles allow the amount of sampling to achieve precision and accuracy in the measurement. V1, V2, V3 are the microvalves used as graphic demonstration.

The switching times of the microvalves for the auto-calibration and sample injection process are fixed in a virtual interface as shown in Fig. 17.

For this microanalyzer, the opto-microfluidic has a total volume of *V* = 1.63 mL, with a total length to the detection cell of *L* = 518 mm and the linear velocity of the fluid is *V_s_* = 20.69 mm/s with an advection time of *t_ad_* = 25 s.

The times were calculated and programmed according to the miniaturized and simplified analytical method, obtaining the following values:

Start time: 25 s; Cycles: 3 (sampling in triplicate); time delay = 2 s (time between samples); tm: 30 s td = 25 s; te = 1.4 s; tr = 25 s; V1 must generate the baseline with an injection time of 1.6 s.

**Fig. 18:**
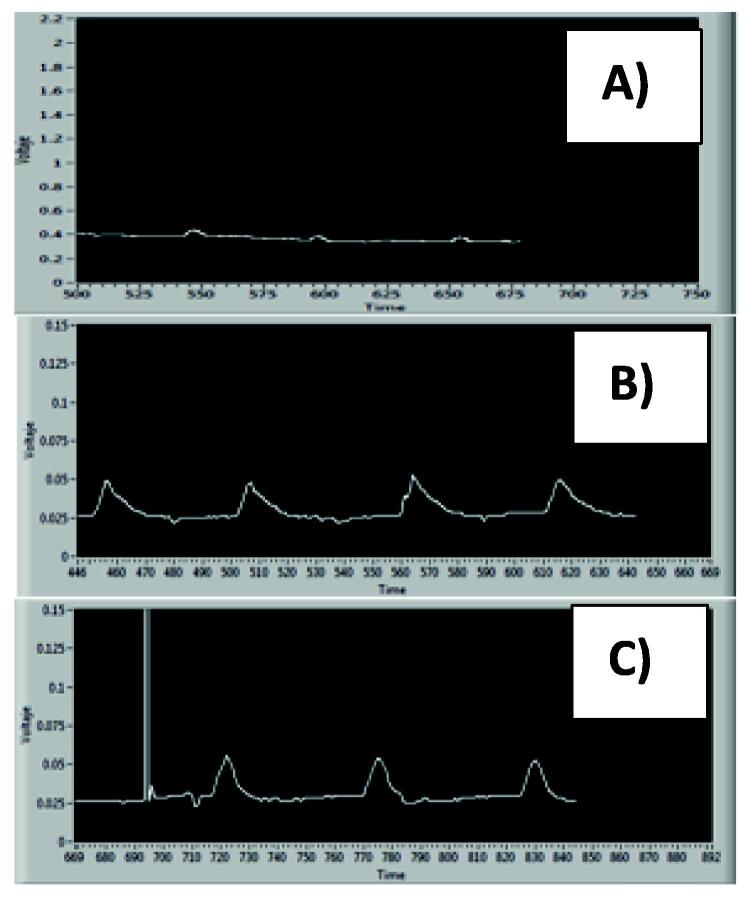
FIAgram for optimal hydrodynamic values. A) Q = 1.5 mL/min, V = 250 µL; B) Q = 3 mL/min, V = 250 µL; C) Q = 3 mL/min, V = 500 µL.

It is important to consider that the volume of the sample and the flow rate must be adjusted to obtain the best signal from the “FIAgram”, see in Fig. 18. The optimal value for the best FIAgram is shown in Fig. 18 C).

This program, could also allow the use of other microfluidic structures with different geometry and dimensions for greater robustness and scaling to the microanalyzer.

### Automated analysis

A control panel was designed for the automated processes based on a virtual instrument program. The design of this virtual instrument is made up of two control windows, where all the necessary operations for the development of the auto-calibration of the microanalyzer were considered. This design was based on a sequential state or event machine architecture, correlating each of them through structures.

1- Set up configuration window

• The *CONFIGURATION* window is where the actions for the configuration of the hydrodynamic system, the detection system and the serial communication port are defined. The section *VALVES*, allows the user to turn the valves on and off and activate only those that intervene in the process.
• The section *PUMPS*, with the virtual knob *FREQUENCY,* allows the user to select the micro-pumps flow rate.
• The section DETECTOR, allows the user to select the filter to use and the scaling of the signal of the detection module.
• In the section called *Port*, the user selects the serial communication port to activate the wireless module and the PC is linking with the electronic control module.
• By pressing the *Set* button once the configuration is done, the user fixes these configuration values ​​in the memory.
• Pressing the *ACQUISITION* button, the user can see the acquisition data in a waveform graph and the *TextBox* in a voltaje/time function, according to the parameters selected. If the user no longer wishes to acquire this signal, the *ACQUISITION* button will have to be pressed again. This process must be done before the microanalysis is carried out.

**Fig. 19:**
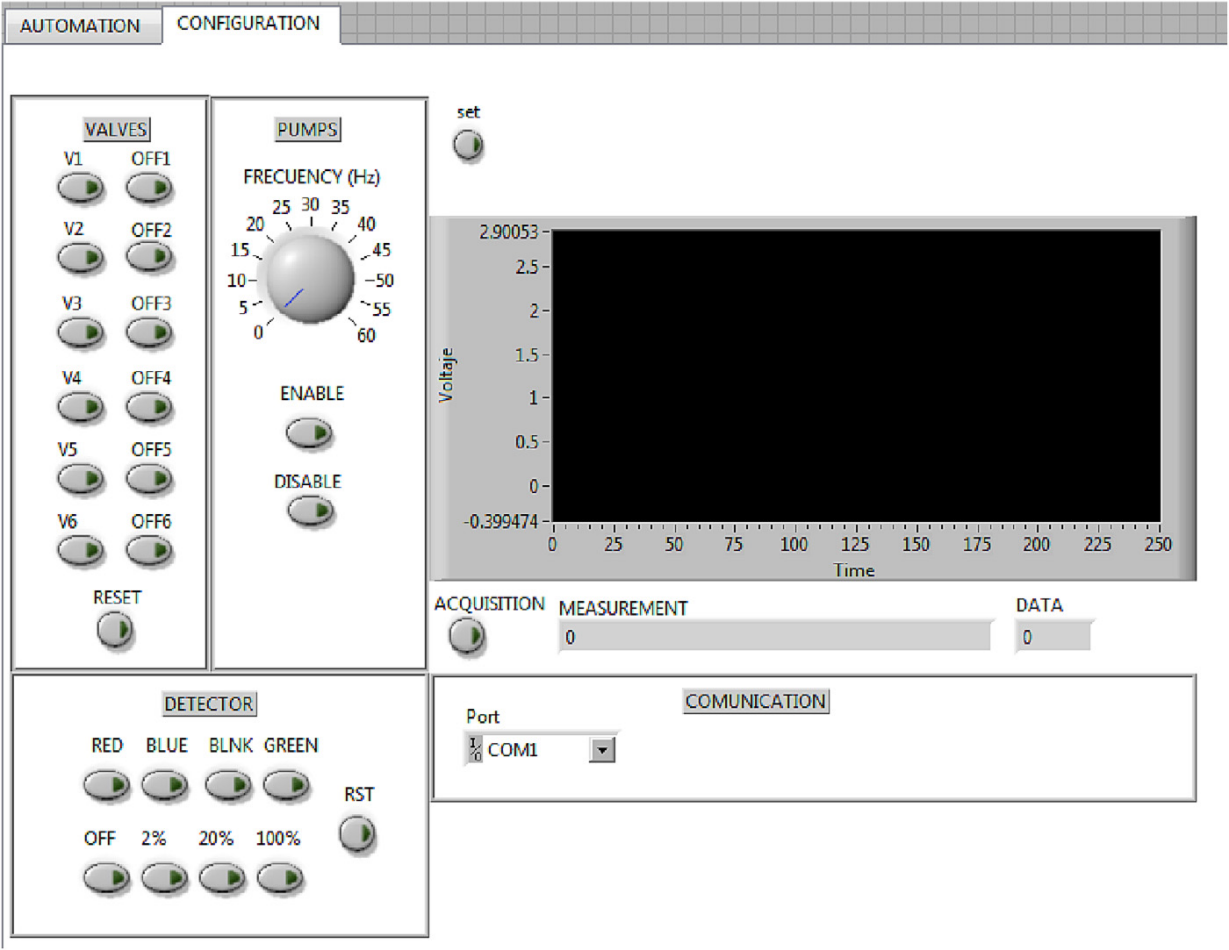
Configuration window for setup.

This configuration window is shown in Fig. 19.

2- Automation analytical processes window

**Fig. 20:**
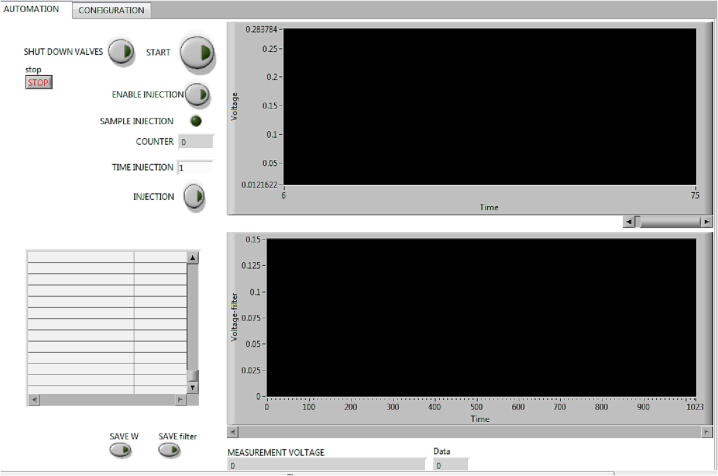
Frontal panel of virtual instrument. Automated window for analytical process.

In the frontal panel (*AUTOMATION* window) there are controls to program or adjust each one of the variables that are necessary to carry out the calibration autonomously, see Fig. 20. These controls are located in a specific order to be activated sequentially one after the other.

• The *START* button, allows the user to start the automation of the analytical process by activating the valves involved in the development of the baseline or transport flow and start the acquisition of the signal generated by the detection system (Sr) from ec. 1. It also resets the injection time *COUNTER* to zero.
• With the *ENABLE INJECTION* button, the user activates the sample injection process, switching the valves involved.
• With the *TIME INJECTION* control, the user programs the injection time in seconds, according to the miniaturized and simplified method proposed.
• Finally, the user executes the sample injection process with the boolean control *INJECTION* during the previously defined time and this is displayed in a numeric indicator *COUNTER* and acquired the analytical signal, (Sa).
• Two waveform graphs are included in the frontal panel, where the user can observe the analytical signal generated in real time (graph above) and the filtered one (graph below).
• It is also possible to save the data in a text file by activating the *SAVE W* button for later processing.
• The user can turn off the valves with the *SHUT DOWN VALVES* control after performing the calibration.

## Validation and characterization

**Fig. 21:**
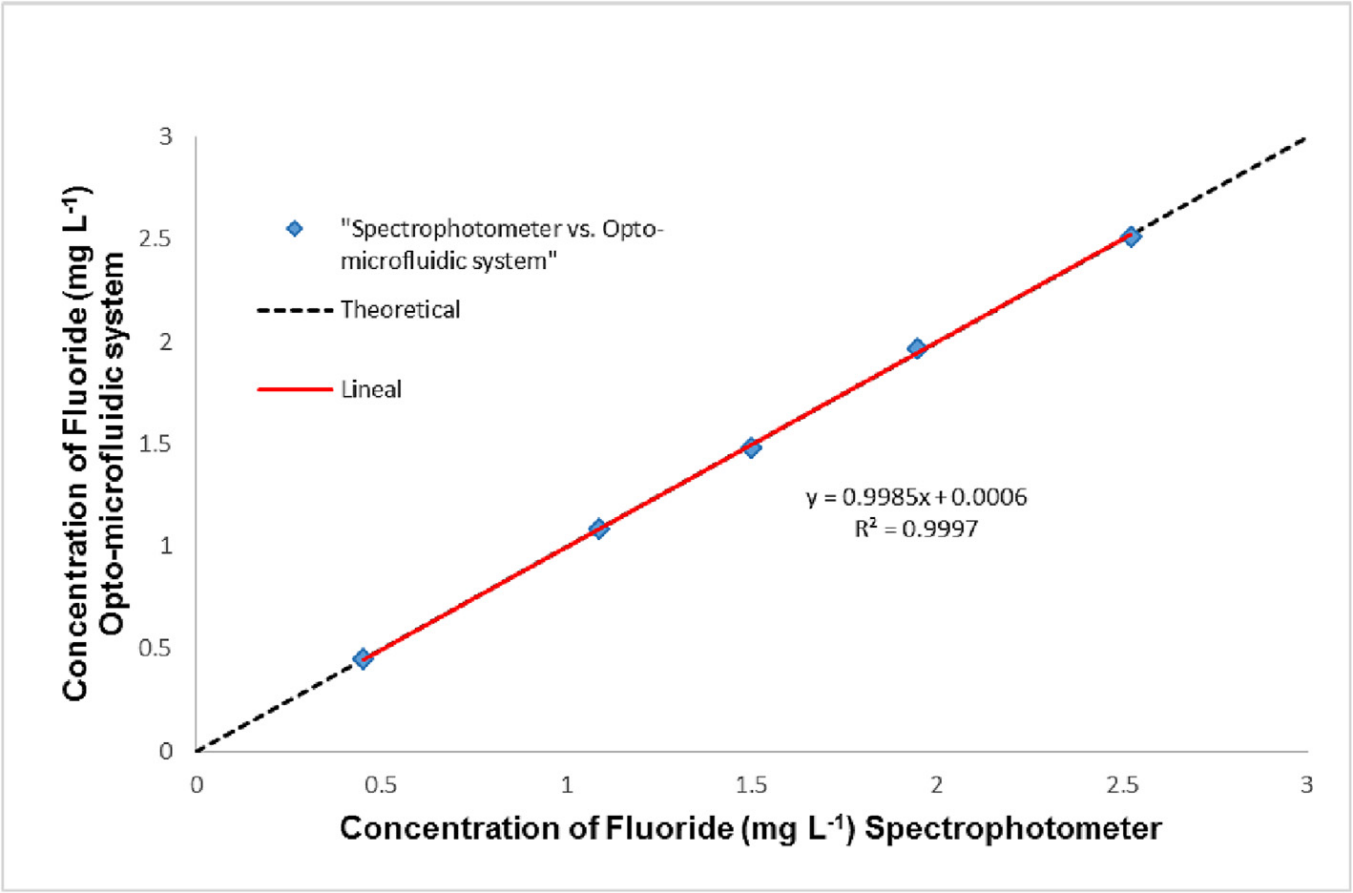
Comparative response between the 3D opto-microfluidics system and the spectrophotometer as a function of the concentration [18].

In auto-calibration processes, standard solutions with fluoride concentration ranging from 0.5 to 2.5 mg L^−1^ were developed. A linear response was obsereved. The equation that describes this linear behavior is A = −0.0473 [F] + 0.2052; R^2^ = 0.9946, a sensitivity of 0.00473 mg L^-1^ a wavelength of 570 nm. For a concentration of 1.5 mg L^-1^, the relative standard deviation RSD calculated was 0.07% (n = 4; 95% confidence). In this application, the detection limit was estimated as 0.5 mg L^-1^ (RSD (%) = 0.07, (n = 4; 95% confidence)). The result was compared with those obtained with a spectrophotometer. Fig. 21 depicts both results, which were plotted against each other. A linear behavior can be observed with a slope close to the unit (0.9997). This confirms traceability between both measurements.

Three groundwater samples with fluoride concentration were automatically analyzed. The samples were taken in deep wells for drinking water in the Lagunera region, which were provided by federal depended National Water Comission, Mexico (CNA). The absorbance measured by the microanalyzer was compared with measurements made previously using a DR/2010 Spectrometer (HASH Co., Col, USA). The comparative results are shown in Table 2. The system response for fluoride in groundwater samples show high accuracy and reproducibility comparable to that obtained with a commercial instrument.

**Table 2:** Comparative fluoride concentration in groundwater samples.

**Fluoride Sample**	**HASH DR/2010 Spectrometer**	**Microanalyzer**	
**Place name**	**mg L^-1^**	**mg L^-1^**	**RSD %**
Water well “Martha”	1.97	1.967	0.3
Water well “Nazareno II”	0.99	0.996	0.53
Water well “Agustin Melgar”	0.53	0.531	0.18

By integrating a hydrodynamic system based on miniaturized actuators for fluid management, the consumption of reagents is significantly reduced, as is the generation of waste. The automation of the injection process controls the amounts of reagents by injecting only the necessary volume, this is because the delivered volume depends on the on–off time of the valve, which can be controlled and measured. The auto-calibration process reduced the consumption of reagents by 50 times compared to the consumption using a peristaltic pump and a modular valve positioner, as shown in Table 3.

**Table 3:** Comparative values of consumption of reagents and waste.

**System**	**SPANDS**	**ZrCl_2_**	**HCl**	**NaF**	**1 calibratoin point**	**TOTAL Calibration** **(waste)**
Peristaltic pump and modular valve positioner	415 µL	21 µL	290 µL	4.16 mL	5 mL	25 mL
Hydrodynamic system with auto-calibration	8.35 µL	417 nL	5.85 µL	83.3 µL	100 µL	500 µL

According to Table 4, the microanalyzer has a consumption of 183.5 mA in standby and 750 mA in operation. Sampling time is 30 s, the battery can perform 330 samples with a frequency of 2 samples per hour. The hydrodynamic system can operate for 7 continuous days.

**Table 4:** Electric parameters of the Microanalizer.

**Device**	**Operation**	**Power**
Driver micropump	Standby	40 mW
	In operation	240 mW
Micropump	Minimum flow rate	18 mW
	Maximum flow rate	71 mW
Valve	Off	10 mW
	On	1 W
Control and acquisition module	On	603 mW
Wireless module	Rx	5 mW
	Tx	100 mW µs^−1^

## Discussion

• The miniaturization and simplification of the analytical method must be carried out as an analysis prior to the operation of the microanalyzer.
• The values of simple volume and injection time are obtained from the dimensions of the opto-microfluidic structure to determine the mass transport and mixing time through the micro-channels towards the detection cell.

## CRediT authorship contribution statement

**Camarillo-Escobedo Rosa:** Conceptualization, Investigation, Writing – original draft, Supervision. **Flores-Nuñez Jorge:** Data curation, Visualization, Writing – review & editing. **García-Muñoz Luis:** Software, Data curation, Validation. **Camarillo-Escobedo Juana:** Methodology, Supervision. **Peña-Dominguez Edgar:** Software, Validation.

## Declaration of Competing Interest

The authors declare that they have no known competing financial interests or personal relationships that could have appeared to influence the work reported in this paper.
